# Efficiency Enhancing Technique for Rod Fiber Picosecond Amplifiers with Optimal Mode Field Matching

**DOI:** 10.3390/mi14020450

**Published:** 2023-02-15

**Authors:** Danni Liu, Xiaojie Mao, Guojiang Bi, Tianqi Li, Dawei Zang, Ninghui Sun

**Affiliations:** 1 Institute of Computing Technology, Chinese Academy of Sciences, Beijing 100190, China; 2 Science and Technology on Solid-State Laser Laboratory, North China Research Institute of Electro-Optics, Beijing 100015, China; 3 The Science and Technology on Inertial Laboratory, School of Instrumentation and Opto-Electronics Engineering, Beihang University, Beijing 100191, China

**Keywords:** rod fiber, picosecond amplifier, mode field matching, fiber coupling

## Abstract

A high power and high quality picosecond laser is crucial in MEMS fabrication regarding micromachines. Optimal seed beam coupling is an important precondition to enhance laser efficiency. However, empirical coupling limits its development. In this paper, the physical parameters related to coupling are determined. The relationships among them are established under optical mode matching constraints to satisfy optimal seed beam coupling. According to a theoretical analysis, the focal length cut-off and the optimal coupling position of the coupling lens are acquired. A maximum transmittance of 87.2% is acquired with a 6 W input seed power in the validation experiment. In further power amplification experiments, a diffraction-limited beam quality is achieved, with M^2^_X_ = 1.111, M^2^_Y_ = 1.017, an optical efficiency of 60.5% and a slope efficiency of 66%, benefiting from the previous theoretical guidance.

## 1. Introduction

The development from machine to micromachine depends on the MEMS fabrication. Etching [1], micro drilling [2], cutting [3] and thickness scanning [4] in MEMS fabrication need high power and a picosecond laser with excellent stability. A picosecond laser system is composed of an optical and a cooling structure, which is involved in stabilizing the laser system. According to the difference in the active regions in the optical structure, picosecond amplification systems can be divided into cryogenic Yb:YAG, thin-disk, Innoslab, single-crystal fiber (SCF), double clad active fiber (DCF) and photonic crystal fiber (PCF) systems. The former three solid-state lasers have the problem of being complicated systems with low stability, beam quality and conversion efficiency. Although the SCF is a simpler system with relatively high stability, beam quality and conversion efficiency compared to other solid-state lasers, it is still inferior to fiber lasers. The DCF system overcomes the above disadvantages of solid-state lasers. However, to satisfy single-mode operation, the mode field area of the DCF is limited to about 700 μm^2^ [5]. The limited mode field area of DCF causes significant nonlinearity and damage under high power operation, which limits output power. Benefiting from an endless single-mode property, PCF can ensure single-mode operation while having a large mode area [6], which effectively controls fiber nonlinearity and potential material damage [7]. So far, the largest reported mode field diameter (MFD) of silica active PCF is 135 μm [8]. Nowadays, PCF, especially large mode area PCF (LMA-PCF), has been proven to be attractive. A comparison of the above different picosecond amplification systems is shown in Table 1.

Rod-type PCF is a kind of LMA-PCF, and is also called rod fiber. It was first proposed for use in ultrashort pulse amplification in 2005 [19]. The rod fiber is suitable for chirped pulse amplification (CPA), divided pulse amplification (DPA) and master oscillator power amplifier (MOPA). Based on the CPA technique, a 100 W average power femtosecond output was generated by a rod fiber amplification system [20]. However, due to the limited stretchability and compressibility of picosecond pulses induced by the narrow spectral bandwidth [21], the CPA technique is unsuitable for picosecond pulse amplification. The DPA technique uses a polarization-controlled free-space delay line or a birefringent crystal to divide and recombine pulses [22]. However, pulse distortion and polarization degradation induced by the pulse division and recombination cause power loss, which limits the efficiency of the DPA system. The MOPA technique has proven to be attractive because the fiber nonlinearity can be controlled effectively. According to the reported studies, rod fiber MOPA systems are developing towards higher power, beam quality and efficiency [23,24].

The optical efficiency enhancement method reported in the literature is mainly based on structure modification, assuming that the seed beam coupling is optimal. In actual experiments, optimal seed beam coupling is an important precondition [25]. However, traditional seed beam coupling depends on experiential adjustment. The empirically optimal seed beam coupling efficiency is easily affected by subjective factors. This study aims to provide the basic theory of optimal seed beam coupling, which guides the physical parameters and the installation position of seed beam coupling devices in actual applications.

In this paper, the theory basis for optimal seed coupling is presented, the related physical parameters are determined and the constraints of these parameters are provided. We begin with determining the optical mode matching condition that satisfies optimal seed beam coupling. A relationship among the related physical parameters can be established under an optimal optical mode matching situation. Based on the constraints of physical applications, the cut-off focal length and the optimal position of the coupling lens are determined. Section 3 describes a seed beam coupling experiment. The theoretical conclusion is verified by changing the single related variables. A passive transmission system is built to acquire the transmittance under different input seed powers, a maximum seed power transmittance of 87.2% is obtained with a 6 W input seed power and the optimal seed beam coupling theory is further enhanced from the perspective of mode field matching. In Section 4, the amplification experiment is implemented and an average power of 101.7 W is generated with a pump power of 162.4 W by using a rod fiber to amplify 6 ps pulses with a 30 MHz repetition rate and a coupled seed power of 5.23 W. The diffraction-limited beam quality with M^2^_X_ = 1.111 and M^2^_Y_ = 1.017 is also obtained. In addition, a maximum optical efficiency of 60.5% and a slope efficiency of 66% are acquired with a rod fiber length of 0.8 m.

## 2. Seed Beam Coupling Theory Based on Optical Mode Field Matching

### 2.1. The Influence Factors of Power Loss

The power loss of seed beam coupling with ultrafast pluses into rod fibers is mainly influenced by two factors: fiber loss and mode field mismatch.

For rod fibers, fiber loss is mainly caused by dissipation from the core to the clad, absorption from the core and reflection from the end face. According to the specification of the rod fibers (aeroGAIN-ROD-PM85), the two end facets of the fiber are coated with an antireflection coating and cut at an angle less than 0.5°. The total reflectivity of the two end facets is lower than 0.3%, and thus can be ignored. According to the instructions of aeroGAIN-ROD-PM85, the core/clad power ratio (CCR) can be expressed as:

(1) $$R_{core/clad}=10\mathrm{lg}\left(\frac{P_{core}}{P_{total}-P_{core}}\right)$$

**Figure 1:**
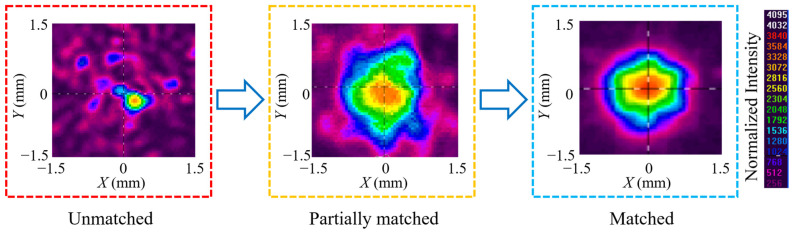
The mode field of coupling 5 W fiber laser into aeroGAINRODPM85 rod fiber.

From Equation (1), when the fiber is in passive operation, the fiber loss from dissipation and absorption can be measured by the CCR. The typical value of the CCR at 1030 nm is about −1 dB and it is relatively stable. Nevertheless, the mode field mismatching is mainly related to the parameters of laser path and coupling lens. It is very sensitive and changeable. If the parameters of the laser path and coupling lens are inappropriate, the power loss will increase significantly to even higher than 50% and the mode field of the passed beam will degrade, as shown in Figure 1. Mode field mismatch is the main influencing factor of power loss.

**Figure 2:**
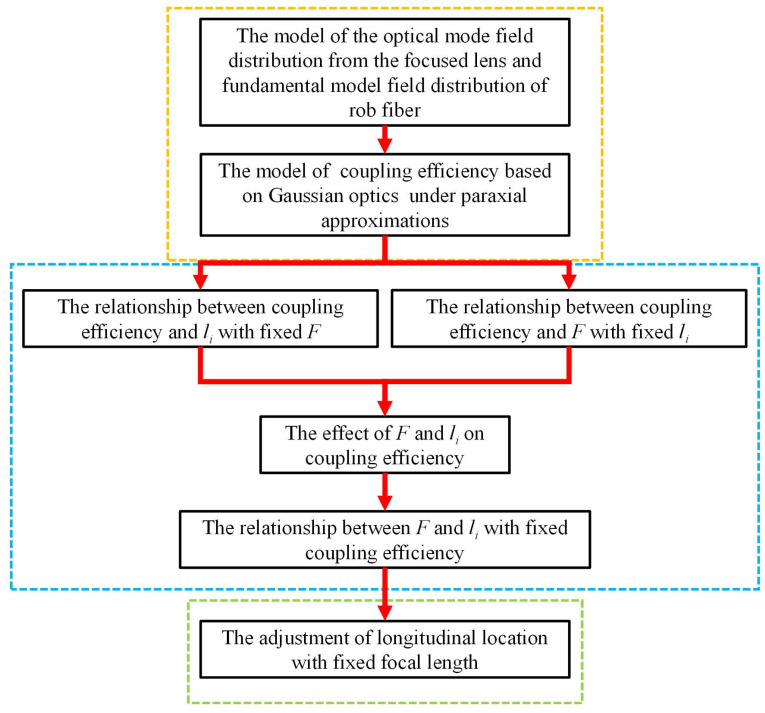
The scheme of the theoretical analysis.

Mode field mismatch can be classified into lateral and longitudinal mismatch. Lateral mismatch is decided by the pitch angle and the lateral position of the lenses. Longitudinal mismatch is decided by the longitudinal position and the focal length of the lenses. Lateral mismatch is easily detectable. Therefore, this study mainly focuses on longitudinal mismatch. In experiments, the longitudinal mismatch can be decreased by adjusting the longitudinal position of the lenses and by choosing lenses with different focal lengths; the theoretical analysis will prove that the former is more effective. The scheme of the theoretical analysis is shown in Figure 2.

### 2.2. Analysis of Optical Mode Field Matching

**Figure 3:**
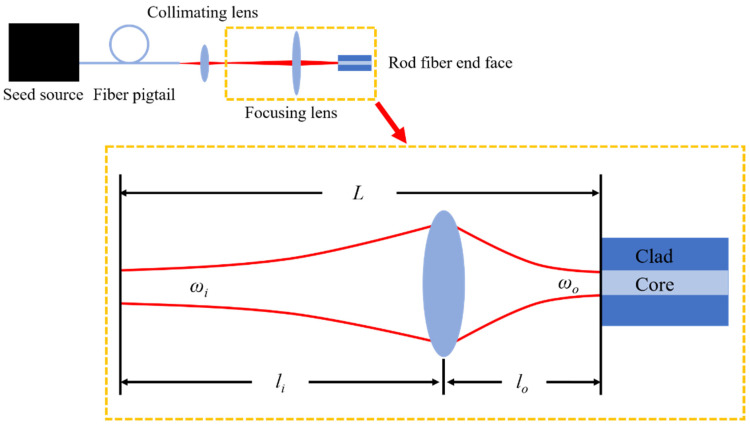
The scheme of lens coupling.

Mode field matching between the incident beam and the rod fiber is mainly measured by the coupling efficiency. The scheme of the coupling system is shown in Figure 3.

In the amplification system, the beam from the seed source is collimated by a collimating lens and focused by a coupling lens into the rod fiber core. In the actual experiment, to reduce the complexity of adjustment, the seed fiber end is placed at the focus point of the collimating lens. Therefore, the position and radius of the collimated beam waist are determined. Therefore, in the subsequent analysis, the collimated beam waist will be the starting point. According to the amplification system in this study, the following conditions can simplify the analysis while ensuring accuracy:

(1) The beam quality of the seed source is diffraction limited (M^2^~1.1), so the seed beam can be approximated as a fundamental mode Gaussian beam.

(2) The length of the laser path (~1 m) is far more than the thickness of the lens (~3 mm), and the diameter of the lens (~20 mm) is also far more than the diameter of the beam on the lens (~1.7 mm), so the lens can be simplified as an ideal thin lens.

The beam waist radius before coupling and the beam radius after coupling are defined as $\omega_{i}$ and $\omega_{o}$, respectively. The distance between the collimated beam waist and the rod fiber end face, the distance between the collimated beam waist and the coupling lens and the distance between the coupling lens and the focused beam waist are defined as L, $l_{i}$ and $l_{o}$, respectively. The focal length of the coupling lens is defined as F. The wavelength of the incident beam is defined as λ.

L, $l_{i}$ and $l_{o}$ satisfy:

(2) $$L=l_{i}+l_{o}.$$

The transformation matrix of the system can be expressed as:

(3) $$T=\left(\begin{array}{cc} A & B\\ C & D \end{array}\right)=\left(\begin{array}{cc} 1 & 0\\ -\frac{1}{F} & 1 \end{array}\right)\left(\begin{array}{cc} 1 & l_{i}\\ 0 & 1 \end{array}\right).$$

The confocal parameter of the input beam is described as:

(4) $$f_{i}=\frac{\pi \omega_{i}^{2}}{\lambda }.$$

The q parameter of the input beam waist satisfies:

(5) $$q_{i}=jf_{i}$$

where j is an imaginary unit. The q parameter after system transformation is described as:

(6) $$q_{o}=\frac{Aq_{i}+B}{Cq_{i}+D}.$$

The absolute value of the real part of $q_{o}$ is the distance between the coupling lens and the output beam waist. The imaginary part of $q_{o}$ is the confocal parameter of the output beam.

(7) l=|Re(qo)|

(8) fo=Im(qo).

The output beam waist radius can be expressed as:

(9) $$\omega =\sqrt{\frac{f_{o}\lambda }{\pi }}.$$

The q parameter of the output beam waist can be described as:

(10) $$q=jf_{o}.$$

The output beam radius on the rod fiber end face can be described as:

(11) $$\omega_{o}=\omega \sqrt{1+{\left(\frac{l_{o}-l}{f_{o}}\right)}^{2}}.$$

The q parameter of the output beam on the rod fiber end face can be expressed as:

(12) $$q_{o}=q+l_{o}-l.$$

The output beam mode field on the rod fiber end face can be described as:

(13) $$U\left(r\right)\propto \exp\left\{-j\left[k\left(l_{o}-l\right)-\arctan\left(\frac{l_{o}-l}{f_{o}}\right)\right]-\frac{jkr^{2}}{2q_{o}}\right\},$$

where k=2π/λ is the wave number of output beam.

Since the rod fiber is in single-mode operation, the rod fiber mode field can be approximated as a Gaussian distribution and described as:

(14) $$U_{P}\left(r\right)\propto \exp\left[-\frac{r^{2}}{{\left(D_{MF}/2\right)}^{2}}\right],$$

where $D_{MF}$ is the mode field diameter (MFD) of the rod fiber.

According to the coupling Gaussian beam into the rod fiber, the ideal condition that the beam axis should align to the rod fiber axis and the rod fiber end face should match the beam waist is satisfied. When the coupled beam waist is located at the rod fiber end face, $l_{o}$ and l satisfy:

(15) $$l_{o}=l.$$

Under this condition, ω, $\omega_{o}$, q and $q_{o}$ satisfy:

(16) $$\omega_{o}=\omega$$

(17) $$q_{o}=q.$$

Therefore, the output beam mode field on the rod fiber end face is reduced to the beam waist mode field:

(18) $$U\left(r\right)\propto \exp\left(-\frac{r^{2}}{\omega^{2}}\right).$$

Under this circumstance, ω is the only factor affecting the mode field matching and ω is related to F and $l_{i}$.

The mode field coupling efficiency can be calculated by the mode field overlap integral [26]:

(19) $$\eta =\frac{{\left|\int_{S}U•U_{P}^{*}dS\right|}^{2}}{\int_{S}{\left|U\right|}^{2}dS•\int_{S}{\left|U_{P}\right|}^{2}dS},$$

where $U_{P}^{*}$ is the complex conjugate of $U_{P}$.

According to the control variable principle, the analysis can be divided into two conditions:

(1) *F* is fixed and *l_i_* is changeable.

**Figure 4:**
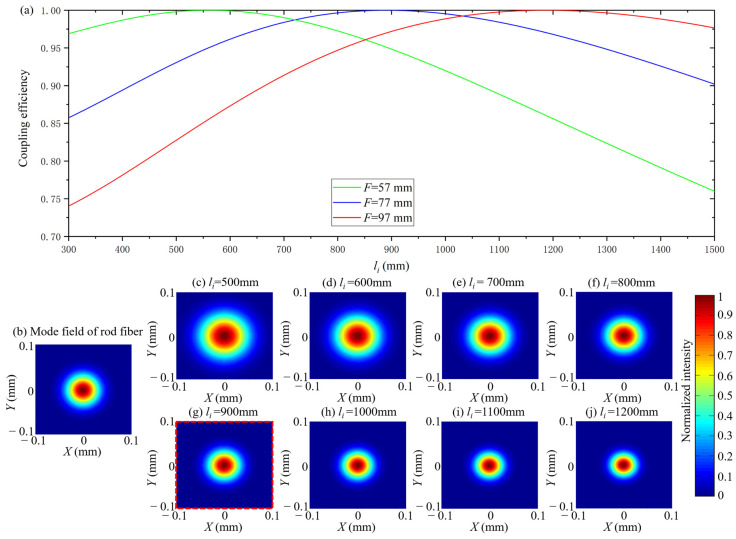
The coupling efficiency as a function of *l_i_* with different *F* when *l*_0_ = *l* (**a**), the mode field of rod fiber (**b**), the mode field of coupled beam when *l_i_* increased from 500 mm to 1200 mm with *F* = 77 mm (**c**–**j**).

The calculation parameters are F=57mm,77mm,97mm, $\omega_{i}=0.4\;\mathrm{mm}$, $D_{MF}=65\;\mu m$ and λ=1030nm. The coupling efficiency as a function of $l_{i}$, and the mode field of the coupled beam under F=77mm and different $l_{i}$ are shown in Figure 4.

According to Figure 4a, as *l_i_* increases, coupling efficiency increases first and then decreases. From Figure 4c to Figure 4j, ω decreases with an increase in *l_i_*. Compared Figure 4b with Figure 4g, $\omega =D_{MF}/2$ when *l_i_ =* 900 mm and *F* = 77 mm.The maximum coupling efficiency is obtained when *l_i_ =* 900 mm and *F* = 77 mm according to Figure 4a. According to these simulation results, the mode field matching is essentially the matching between ω and $D_{MF}/2$. Under different values of F, there always exists an *l_i_* which can achieve the maximum coupling efficiency of 1 when $\omega =D_{MF}/2$. When F is larger, the corresponding *l_i_* is larger to get maximum coupling efficiency. The coupling efficiency exceeds 0.975 in a certain range (±200 mm) near the value of *l_i_* which results in the maximum coupling efficiency, which means that the effect of *l_i_* on ω was not significant when *F* is fixed.

(2) *l_i_* is fixed and *F* is changeable.

**Figure 5:**
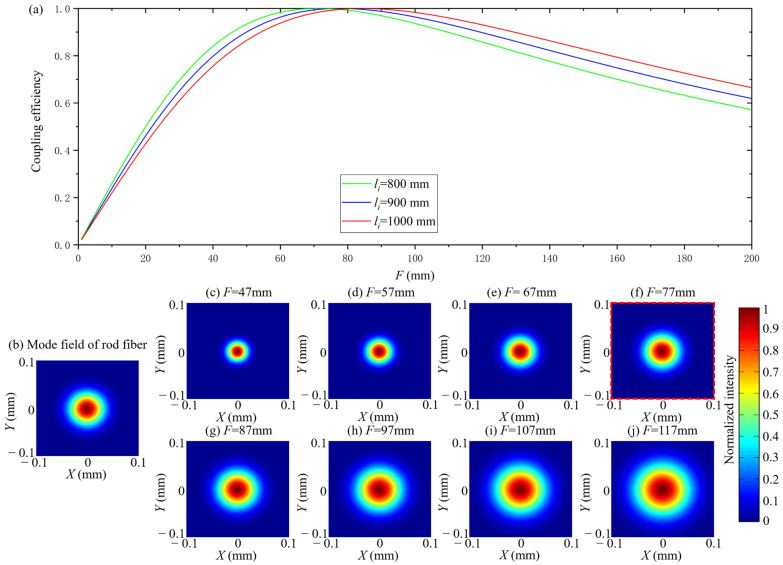
The coupling efficiency as a function of *F* with different *l_i_* when *l*_0_ = *l* (**a**), the mode field of rod fiber (**b**), the mode field of coupled beam when *F* increased from 47 mm to 117 mm with *l_i_* = 900 mm (**c**–**j**).

The calculation parameters are *l_i_* = 800 mm, 900 mm, 1000 mm, $\omega_{i}=0.4\;\mathrm{mm}$, $D_{MF}=65\;\mu m$ and λ=1030nm. The coupling efficiency as a function of F and the mode field of the coupled beam when *l_i_* = 900 mm at different values of F are shown in Figure 5.

According to Figure 5a, as F increases, the coupling efficiency increases first and then decreases. From Figure 4c to Figure 4j, the ω increases with an increasing F. Compared Figure 5b with Figure 5g, $\omega =D_{MF}/2$ when *l_i_* = 900 mm and F=87mm.The maximum coupling efficiency is obtained when *l_i_* = 900mm and F=87mm according to Figure 5a. These simulation results show that the mode field matching is essentially the matching between ω and $D_{MF}/2$ once again. Under different values of $l_{i}$, there is always an F that can achieve the maximum coupling efficiency of 1 when $\omega =D_{MF}/2$. When $l_{i}$ is larger, the corresponding F is larger to get maximum coupling efficiency. The range of coupling efficiency changes significantly when F is increased from 0 mm to 200 mm, which means that the effect of F on ω is significant when $l_{i}$ is fixed. 

In summary, to acquire the optimum mode field matching, the coupled beam waist must be located at the rod fiber end face and the beam waist diameter must be equal to the MFD. The values of $l_{i}$ and F for optimal mode field matching and the corresponding value of $l_{o}$ can be calculated by these two conditions.

### 2.3. Seed Beam Coupling Theory

To further analyze the relation between $l_{i}$ and F quantitatively, the following deduction is carried out.

Combining Equations (3), (5), (6) and (8), an equation which contains $l_{i}$, F, $f_{i}$ and $f_{o}$ can be acquired:

(20) $$\frac{f_{i}}{f_{o}}F^{2}-f_{i}^{2}={\left(F-l_{i}\right)}^{2}.$$

Substituting Equations (4) and (9) into Equation (20), the relation between $l_{i}$ and F can be expressed as:

(21) $$l_{i}=F\mp \frac{\omega_{i}}{\omega }\sqrt{\left(F-\frac{\pi \omega_{i}\omega }{\lambda }\right)\left(F+\frac{\pi \omega_{i}\omega }{\lambda }\right)}.$$

According to Equation (21), $l_{i}$ can be a real number only if $F\geq \pi \omega_{i}\omega /\lambda$. Therefore, the lower bound of F satisfies:

(22) $$F_{c}=\frac{\pi \omega_{i}\omega }{\lambda }.$$

When $F\geq F_{c}$, there are two conditions that should be considered:

(1) Choosing the plus sign, i.e., $l_{i}\geq F$. Under this condition, the input beam waist is located at or in front of the coupling lens focus.

(2) Choosing the minus sign, i.e., $l_{i}\leq F$. Under this condition, the input beam waist is located at or behind the coupling lens focus. In addition, when $\omega_{i}>\omega$, $l_{i}<0$ may occur, which means that the input beam waist is located behind the coupling lens. When $l_{i}=0$, the corresponding F can be expressed as:

(23) $$F=\frac{\omega_{i}}{\sqrt{\omega_{i}^{2}-\omega^{2}}}F_{c}.$$

Therefore, the relation between $l_{i}$ and F can be further expressed as:

(24) $$l_{i}=\left\{ \begin{array}{l} F+\frac{\omega_{i}}{\omega }\sqrt{\left(F-F_{c}\right)\left(F+F_{c}\right)},\;\left(F\geq F_{c}\;\mathrm{and}\;F\leq l_{i}\right)\\ F-\frac{\omega_{i}}{\omega }\sqrt{\left(F-F_{c}\right)\left(F+F_{c}\right)},\;\left(F\geq F_{c}\;\mathrm{and}\;F\geq l_{i}\right) \end{array}\right..$$

**Figure 6:**
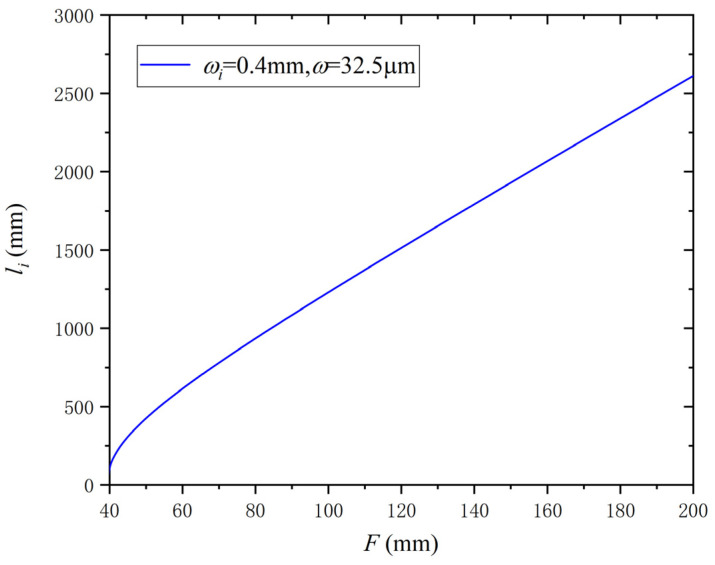
*l_i_* as a function of *F*.

When $\omega_{i}\leq \omega$, the beam is expanded. When $\omega_{i}>\omega$, the beam is focused. It is easy to calculate the values of F and $l_{i}$ which satisfy the maximum coupling efficiency and analyze the relation between F and $l_{i}$ by Equation (24). The analysis in this study satisfies the conditions of $\omega_{i}>\omega$ and $F\leq l_{i}$. Therefore, in the following analysis, the first equation in Equation (24) will be used. The calculation parameters are $\omega_{i}=0.4\;\mathrm{mm}$, ω=32.5 μm and λ=1030nm. $l_{i}$ as a function of F is shown in Figure 6.

According to Figure 6, *l_i_* increases as *F* increases, but *l_i_* rises faster than *F*. This further illustrates the above conclusion that the effect of *F* on the coupling efficiency is more significant than that of *l_i_*.

Through the above theoretical analysis, the optimal seed beam coupling should satisfy the following conclusions:

(1) The coupling lens focal length should satisfy *F* ≥ *F_c_*.

(2) When the focal length is determined, the *l_i_* of coupling lens should satisfy Equation (24).

## 3. Experimental Verification of Seed Beam Coupling

The seed source is a fiber picosecond system, the wavelength is 1030 nm, the output average power is from 0 W to 6 W, the M^2^ value is 1.1 and the MFD of the output fiber is 25 μm. The seed beam is collimated by a lens with a 15 mm focal length and the waist radius of the seed beam after collimating is 0.4 mm. The rod fiber used in the experiment is the aeroGAIN-ROD-PM85 produced by NKT Photonics. The MFD of the rod fiber is 65 μm, the length of the rod fiber is 803 ± 2 mm and the NA of the core of the rod fiber is about 0.006. According to the theoretical analysis, F=77mm, *l_i_* = 900 mm and $l_{o}=l=82.33\;\mathrm{mm}$ were chosen as the laser path parameters for the experiment.

### 3.1. Verification of Optimal Seed Coupling Theory

**Figure 7:**
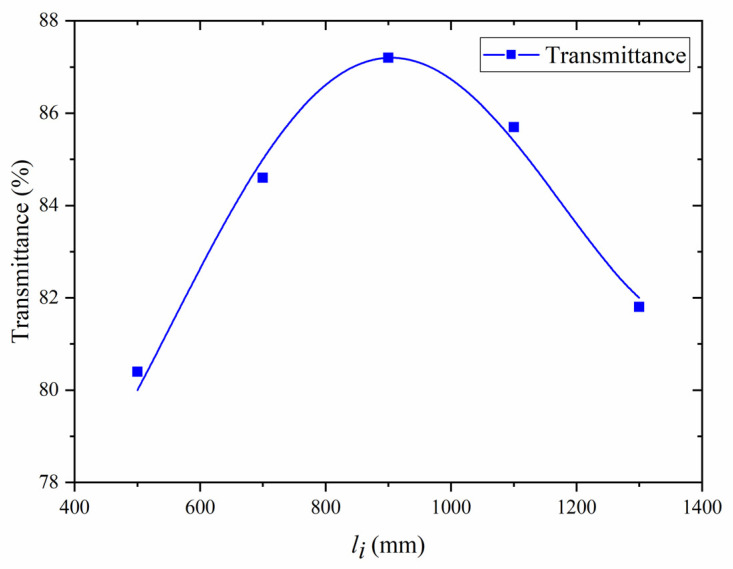
The seed power transmittance when *l_i_* = 500 mm, *l_i_* = 700 mm, *l_i_* = 900 mm, *l_i_* = 1100 mm and *l_i_* = 1300 mm.

In the experiment, the seed power transmittance as a function of $l_{i}$ with fixed F was obtained. According to Figure 7, the maximum transmittance was acquired at *l_i_* = 900 mm. The transmittance increases before *l_i_* = 900 mm and drops after *l_i_* = 900 mm. In addition, the growth is faster than the drop. Although the maximum transmittance at *l_i_* = 900 mm is 87.2% due to fiber loss, the characteristics of the transmittance curve in the figure are consistent with the blue curve in Figure 4, which verifies the correctness and effectiveness of the theoretical analysis.

### 3.2. Seed Beam Coupling of an Amplification System

**Figure 8:**
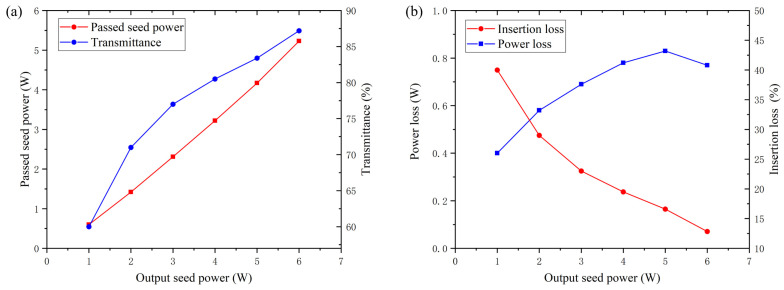
Transient seed power and the transmittance as function of the output seed power (**a**) and power loss and insertion loss as function of the output seed power (**b**).

Under optimal seed beam coupling, to measure the seed power passing through the rod fiber, the transmittance and the coupled mode field under different output seed powers, the output seed power was gradually increased from 1 W to 6 W. The transient seed power and the transmittance with an increasing output seed power is shown in Figure 8a. Meanwhile, the power loss and the insertion loss with an increasing output seed power is shown in Figure 8b.

According to Figure 7a, the transient seed power and the transmittance rise with the increase in the output seed power; the maximum transmittance of 87.2% was acquired at an output seed power of 6 W and the corresponding transient seed power was 5.23 W. From Figure 8b, the power loss increases first and reaches a maximum at 0.83 W at an output seed power of 5 W, then reduces with the further increase in output seed power. The insertion loss reduces with the increase in output seed power. There are two phenomena that need to be explained:

(1) The transmission rate exceeds 44%.

If the mode field of the seed beam waist and fiber core were fully matching, according to the instructions of the rod fiber, when the fiber is in passive operation, the power passing through the fiber core should be 44% of the total power. However, the results of this experiment show that the transmission rate exceeds 44%. The explanation is as follows. In this experiment, an aperture was not used to separate the power passing through the fiber core. Therefore, due to the limited optical field constraining ability of the rod fiber clad, the transmission power was composed of two parts of power. The first part is the power passing through fiber core and the second part is the power passing through the fiber clad. Although the transmission power consists of the power passing through the core and the clad, the proportion of the transmission power still cannot reach 100% of the output seed power, which means that the coupling still has mode field mismatch and the rod fiber has an absorption at 1030 nm.

(2) The insertion loss decreases with an increase in output seed power.

**Figure 9:**
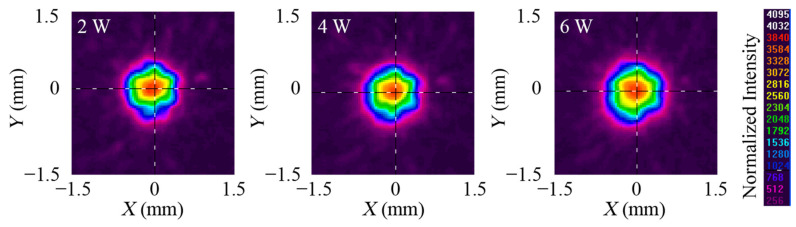
Variation in the coupled spots at output seed powers of 2 W, 4 W and 6 W.

The reason for the variation in the insertion loss is that the seed beam mode field changes with the increase in output seed power and gradually matches the rod fiber mode field. The coupling mode fields at 2 W, 4 W and 6 W are shown in Figure 9; with the increase in output seed power, the mode field distribution of the transient beam has a tendency to be round, which confirms the above explanation.

Based on the experimental results and analysis, the theoretical analysis method has the ability to calculate the optimal mode field matching and enhance the coupling efficiency in experiments, and in the subsequent amplification experiments, 6 W will be selected as the output seed power.

## 4. Further Power Amplification Application

**Figure 10:**
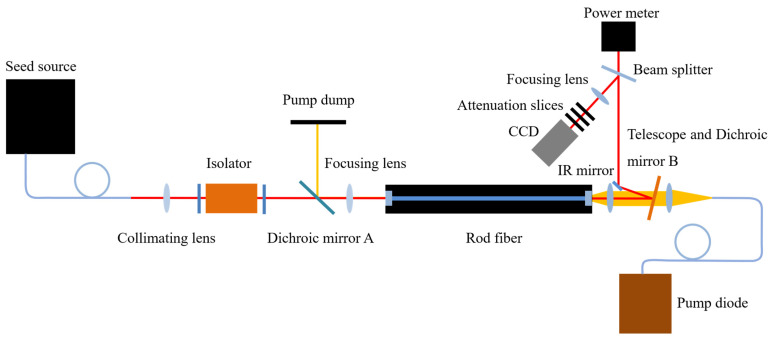
The scheme of the rod fiber amplification system.

A scheme of the rod fiber amplification system is shown in Figure 10. For the convenience of adjustment, a counter-pumped structure was used in the experiment. The seed beam passes through a collimating lens (*F* = 15 mm), an isolator, dichroic mirror A (45° AR at 1030 nm and HR at 976 nm) and a coupling lens (*F* = 77 mm), respectively, then it is coupled to the rod fiber from the front-end. The pump beam passes through a telescope (both lenses are *f* = 15 mm) and dichroic mirror B (0° AR at 940 nm and HR at 1030 nm), respectively, then it is coupled to the rod fiber from the back-end. The infrared (IR) output is reflected into the power meter by dichroic mirror B and an IR mirror. The function of the beam splitter is to separate out a beam with low-power and reflect it into the CCD to observe the output spot. The seed source is a picosecond fiber laser system with a 6 W maximum average power, a central wavelength of 1030.24 nm, an autocorrelation pulse duration of about 5 ps, a repetition rate of 30 MHz, a spectral bandwidth of about 1.2 nm and an M^2^ value of about 1.1. The MFD of the seed fiber is 25 μm. The central wavelength and the maximum output power of the pump diode are 976 nm and 200 W, respectively. The rod fiber is an aeroGAIN-ROD-PM85, produced by NKT Photonics. The MFD is 65 μm and length is 803 ± 2 mm. The two end caps are coated with antireflective film in order to decrease the Fresnel reflection loss. The rod fiber is clamped by a V-groove and sealed by a thermally conductive adhesive. The V-groove and pump diode are cooled by water at 20 °C.

**Figure 11:**
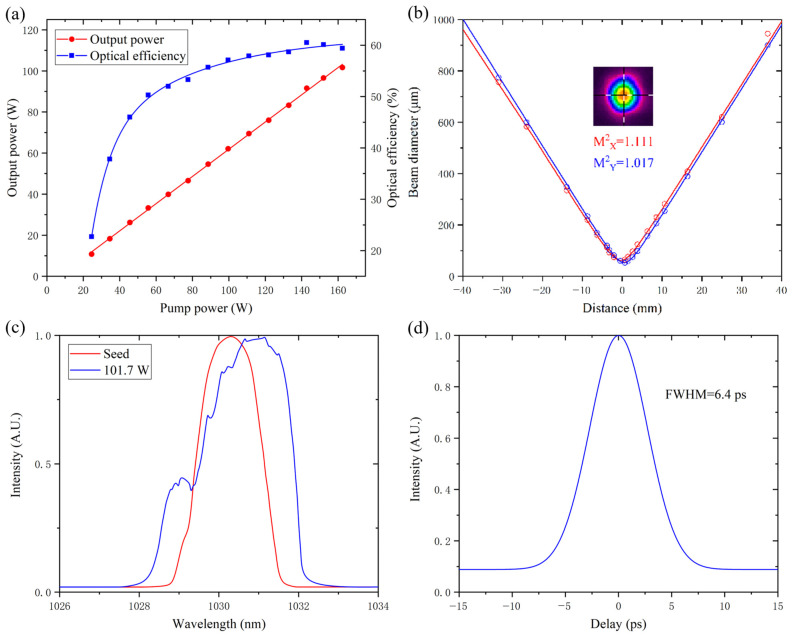
Output power and optical efficiency as a function of pump power (**a**); M^2^ measurement at 101.7 W (**b**); optical spectrum from seed and 101.7 W output (**c**); autocorrelation signal of output pulses at 101.7 W (**d**).

According to the 87.2% transmittance with 6 W output seed power, a coupled seed power of 5.23 W is obtained. When the pump power was increased to 162.4 W, the output power and the optical efficiency as a function of pump power were measured and are shown in Figure 11a. A maximum optical efficiency of 60.5% was achieved with a 142.8 W pump power, and the corresponding output power was 91.6 W. When the pump power reached 162.4 W, a maximum output power of 101.7 W and a peak power of 500 kW were obtained; however, the corresponding optical efficiency was reduced to 59.4%. The slope efficiency of the amplifier was about 66.3%. In addition, the amplifier was unsaturated; however, considering that increasing the pump power may cause damage to the rod fiber and optical efficiency to decline, the pump power was not increased further in the experiment.

The beam diameters in the horizontal and vertical directions are subtly different, as shown in Figure 11b, and the M^2^ values in the two directions are M^2^_X_ = 1.111 and M^2^_Y_ = 1.017 at 101.7 W, respectively, based on the D4σ method. Therefore, near diffraction-limited beam quality is acquired by this amplification system.

The spectral bandwidth of the seed is 1.2 nm (full-width at half-maximum, FWHM), but it was broadened to 1.8 nm at 101.7 W due to the self-phase modulation (SPM), as shown in Figure 11c. Attributed to the large mode area of the rod fiber, the maximum peak power of the output pulses is far below the threshold for stimulated Raman scattering (SRS); no SRS can be observed in the optical spectrum of the IR output. As shown in Figure 11d, the autocorrelation pulse width was measured as 6.4 ps (FWHM) at 101.7 W. The time-bandwidth product is 2.57, so the amplified pulses are chirped.

## 5. Conclusions

In summary, to obtain a high coupling efficiency of the seed beam, thus enhancing the laser output power and beam quality of the rod fiber amplification system, a theoretical framework is established. The optical mode matching condition to satisfy optimal seed coupling is determined. The relationships among the main affecting factors of the coupling efficiency are determined. Among numerous variables, the focal length and position of the coupling lens are determined as the key variables for optimal seed coupling, and the cut-off focal length and the optimal position of the coupling lens are acquired.

According to the seed beam coupling experiment, the theoretical derivation is verified under single variable control. A passive transmission system is built and a maximum transmittance of 87.2% is obtained with an input seed power of 6 W. The optimal seed beam coupling theory is further enhanced considering the mode field mismatching.

The rod fiber amplification system achieves an average power of 101.7 W with a coupled seed power of 5.23 W and a pump power of 162.4 W. The beam quality of the amplification system is diffraction limited, with M^2^ values in the horizontal and vertical directions of 1.111 and 1.017, respectively. A maximum optical efficiency of 60.5% and a slope efficiency of 66% are achieved with a rod fiber length of 0.8 m. Under the optimal mode field matching, the rod fiber amplification system obtains high beam quality and amplification efficiency, which both show good prospects and feasibility in MEMS fabrication.

## Figures and Tables

**Table 1:** Comparison of different picosecond amplification systems.

Type	Stability		Optical Efficiency	M^2^ Beam Quality
	Optical Structure	Cooling Structure		
Cryogenic Yb:YAG crystal [9,10]	Simple coupling system	Liquid nitrogen cooling	≤20%.	1.1–1.5
Thin-disk [11,12]	Complicated coupling system	Heat sink with water cooling	≤50%.	1.1–1.5
Innoslab [13,14,15]	Complicated coupling system	Heat sink with water cooling	≤40%.	1.1–1.5
SCF [16]	Simple coupling system	Heat sink with water or passive cooling	50–60%.	≤1.2
DCF [17]	Fiber coupler	Passive cooling	≥70%.	≤1.1
PCF [18]	Simple coupling system or fiber coupler	Heat sink with water cooling	≥60%.	≤1.2

## Data Availability

Not applicable.
